# Hepatitis C virus-specific cellular immune responses in individuals with no evidence of infection

**DOI:** 10.1186/1743-422X-9-76

**Published:** 2012-03-28

**Authors:** Yves Rivière, Thomas Montange, Geneviève Janvier, Caroline Marnata, Ludovic Durrieu, Marie-Laure Chaix, Maria Isaguliants, Odile Launay, Jean-Louis Bresson, Stanislas Pol

**Affiliations:** 1 Laboratoire d'Immunopathologie Virale, Institut Pasteur; and CNRS URA 3015, 25 rue du Dr Roux, 75015 Paris, France; 2 EA 3620, Université Paris-Descartes; and Laboratoire de Virologie, CHU Necker-Enfants Malades, AP-HP, Paris, France; 3 Institute for Microbiology, Tumor and Cell Biology, Karolinska Institutet, 17177 Stockholm, Sweden; 4 Université Paris-Descartes; AP-HP, Hôpital Cochin, CIC de Vaccinologie Cochin-Pasteur Inserm CIC BT505, Paris, France; 5 Centre d'investigation clinique, Hôpital Necker-Enfants Malades, Université Descartes, Paris, France; 6 Unité d'Hépatologie, Hôpital Cochin, AP-HP; INSERM U1016; and Université Paris-Descartes, Paris, France

**Keywords:** HCV, Prevalence, Proliferation, Elispot, Inapparent infection

## Abstract

The detection of hepatitis C virus (HCV)-specific T cell responses in HCV-uninfected, presumably unexposed, subjects could be due to an underestimation of the frequency of spontaneously resolving infections, as most acute HCV infections are clinically silent. To address this hypothesis, HCV-specific cellular immune responses were characterized, in individuals negative for an HCV PCR assay and humoral response, with (n = 32) or without (n = 33) risk of exposure to HCV. Uninfected volunteers (n = 20) with a chronically HCV-infected partner were included as positive controls for potential exposure to HCV and HCV infection, respectively. HCV-specific T cell responses in freshly isolated peripheral blood mononuclear cells were studied *ex vivo *by ELISPOT and CFSE-based proliferation assays using panels of HCV Core and NS3-derived peptides. A pool of unrelated peptides was used as a negative control, and a peptide mix of human cytomegalovirus, Epstein-Bar virus and Influenza virus as a positive control. Overall, 20% of presumably HCV-uninfected subject tested had detectable T-cell responses to the virus, a rate much higher than previous estimates of HCV prevalence in developed countries. This result would be consistent with unapparent primary HCV infections that either cleared spontaneously or remained undetected by conventional serological assays.

## Background

Hepatitis C virus (HCV) is a positive stranded RNA virus belonging to the *Flaviviridæ *family. HCV replicates mainly in the liver, and approximately 70% of infected persons fail to spontaneously clear the virus, progressing to chronic infection. HCV infection is defined as the detection of specific antibodies in the serum (by two different screening assays), with or without detectable HCV-specific RNA which reflects ongoing or resolved infection, respectively. An estimated 170 million persons worldwide are infected by HCV.

Three sets of data challenge current estimates of the proportion of HCV-infected patients that become chronic carriers [1]. Firstly, HCV-specific T lymphocytes are found in the blood of donors who do not meet current criteria for HCV infection, displaying a weak or restricted specific antibody response labeled as an 'indeterminate pattern' in the recombinant immunoblot confirmation assay [2]. Secondly, the clearance of HCV has been reported in individuals without detectable seroconversion [3]. Thirdly, the disappearance of circulating anti-HCV antibodies some two decades after an accidental inoculation has been documented in patients who spontaneously resolve their infection, although HCV-specific CD4^+ ^and CD8^+ ^T-cell responses were detectable [4].

Thus, as most acute HCV infections are clinically silent, the detection of a virus-specific T cell response in healthy presumably unexposed subjects who do not meet current criteria for a previous HCV infection can be due to preceding silent spontaneously resolved HCV infection, the frequency of which is apparently underestimated [5,6]. Viral infection in such individuals would have produced enough viral immunogen to prime T cells, but yet not enough to prime an IgG B cell response that could be detected by the available commercial assays [3,7]. If confirmed, such an hypothesis could change our views concerning the epidemiology and physiopathology of HCV infections. An alternative hypothesis could be the existence of T cell epitope cross-reactivity between other pathogens or common antigens present in the general population and HCV as previously reported [8-10]. These two hypotheses are not mutually exclusive.

To investigate such possibilities, HCV-specific cellular immune responses were characterized in uninfected individuals (UI) where neither the presence of HCV RNA nor that of anti-HCV antibody had previously been detected. Exposed uninfected volunteers (EUI) and their chronically HCV-infected (CI) sexual partners were included in this study as positive controls for HCV exposure and infection, respectively.

## Results

We assembled three cohorts of individuals that differed in their history with respect to potential exposure to HCV, and HCV infection. The first group comprised apparently noninfected, nonexposed volunteers (UI), although about half of these were at risk of exposure to the virus. Exclusion factors for exposure to HCV [11] were: professional exposure, drug abuse, blood transfusion or injection of blood products, sexually transmitted diseases, incarceration, alcoholism, dialysis, endoscopy, acupuncture, mesotherapy, invasive cosmetic treatment, piercing, tattooing, sexual exposure, familial exposure, and hospitalization or outpatient treatment in a developing country. A second group consisted of individuals who remained uninfected despite repeated exposure (EUI), who were in fact exposed through their sexual contact with a member of the third group: their long-standing chronically-infected (CI) partners.

### HCV-specific responses

#### Proliferative responses

**Figure 1:**
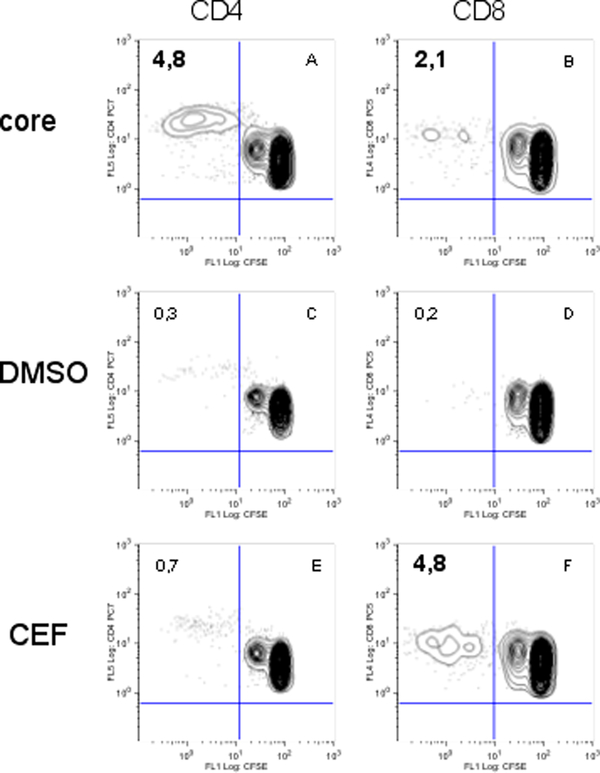
**example of proliferation for PBMC of volunteer EFS 20**. Dot-plots show the percentage of proliferative CD8 ^+ ^(FL1/FL4) or CD4 ^+ ^(FL1/FL5) - T cells. The number in the upper left panel stands for the percentage of CD4^+ ^or CD8^+ ^proliferative cells among the total CD4^+ ^or CD8^+ ^-T lymphocyte population, respectively, in the absence (DMSO) or the presence of Core or CEF antigens. The positive responses are in bold characters.

HCV-specific proliferative T cell responses were tested in 62 of the 65 uninfected volunteers (UI). Among these, 2 were positive for Core, and 4 for NS3 (Table 1). The response directed against Core involved both CD4 and CD8 populations for volunteer EFS20 who had no known risk of HCV exposure (Figure 1, panels A & B, and Table 2), and a CD4 population for EFS 11 (at risk) (Table 3). For NS3, a response involved the CD4 population (COC 13, Table 2, and EFS14, Table 3), the CD8 population (EFS 21, Table 3) or both CD4 and CD8 populations (EFS 24, Table 3). All three EFS donors were at risk of HCV exposure, in contrast to risk-free volunteer COC 13. None of the supposedly uninfected (UI) volunteers was found to be positive for both NS3 and Core.

**Table 1:** Proliferative and Elispot responses in chronically HCV-infected, exposed and uninfected volunteers

		PROLIFERATION			ELISPOT		
**VOLUNTEERS**		**CORE**	**NS3**	**CEF**	**CORE**	**NS3**	**CEF**

	All	2/61 (3)^4^	4/62 (6)	24/62 (39)	8/65 (12)	0/59 (0)	31/58 (53)
	
**UI^1^**	No risk	1/29 (3)	1/30 (3)	10/30 (33)	4/33 (12)	0/29 (0)	13/28 (46)
	
	At risk	1/32 (3)	3/32 (9)	14/32 (44)	4/32 (13)	0/30 (0)	18/30 (60)

**EUI^2^**		0/17 (0)	0/17 (0)	5/17 (29)	6/20 (30)	1/11 (9)	6/11 (55)

**CI^3^**		3/17 (18)	2/16 (12,5)	9/16 (56)	8/20 (40)	2/6 (33)	3/6 (50)

1: Uninfected individuals (UI); 2: Exposed uninfected partners (EUI) of, 3: chronically HCV-infected (CI) individuals. 4: number positive/total number tested (percent). Control antigen (CEF) : There were no statistical difference in the frequencies of proliferative or elispot responses to CEF when comparing each group 2 by 2. HCV antigens : There were no statistical difference in the frequencies of proliferative response to both Core and NS3 when comparing each group 2 by 2; The frequencies of Elispot response to Core were higher in the CI group compared to UI no risk (*p *= 0.04) and to UI at risk (*p *= 0.04). The frequencies of Elispot response to NS3 were higher in the CI group compared to UI no risk (*p *< 0.03) and to UI at risk (*p *< 0.03).

**Table 2:** Responses in uninfected volunteers with no known risk of exposure to HCV

I.D	Sex	Proliferation*			Elispot**		
		
		Core	NS3	CEF	Core	NS3	CEF
**CIC 09**	F	(-)	(-)	(-)	(-)	(-)	15

**CIC 16**	M	(-)	(-)	(-)	24	bgd	bgd

**CIC 17**	M	ND	ND	ND	9	ND	ND

**CIC 23**	M	ND	ND	ND	(-)	ND	ND

**CIC 53**	M	(-)	(-)	(-)	(-)	(-)	(-)

**EFS 04**	F	(-)	(-)	6 (T8)	(-)	(-)	252

**EFS 05**	F	(-)	(-)	(-)	(-)	(-)	(-)

**EFS 08**	F	bgd	bgd	bgd	(-)	(-)	(-)

**EFS 10**	F	(-)	(-)	7 (T8)	(-)	(-)	(-)

**EFS 16**	M	(-)	(-)	bgd	(-)	(-)	17

**EFS 18**	F	(-)	(-)	(-)	(-)	(-)	(-)

**EFS 20**	F	9 (T8), 16(T4)	(-)	19 (T8)	18	(-)	9

**EFS 22**	F	(-)	(-)	(-)	(-)	(-)	8

**COC 01**	M	ND	ND	ND	(-)	(-)	ND

**COC 02**	F	(-)	(-)	(-)	(-)	(-)	(-)

**COC 03**	F	(-)	(-)	(-)	(-)	ND	ND

**COC 04**	M	(-)	(-)	14 (T8)	(-)	(-)	9

**COC 05**	M	(-)	(-)	(-)	(-)	(-)	(-)

**COC 06**	M	(-)	(-)	(-)	(-)	(-)	16

**COC 07**	M	(-), bgd	(-), bgd	20 (T8)	(-)	(-)	20

**COC 08**	F	(-)	(-)	5 (T8)	(-)	(-)	(-)

**COC 09**	F	(-), bgd	(-), bgd	16 (T8)	(-)	(-)	24

**COC 10**	M	(-)	(-), bgd	(-)	(-)	(-)	34

**COC 11**	M	(-)	(-)	(-)	(-)	(-)	(-)

**COC 12**	F	(-)	(-), bgd	(-)	(-)	(-)	(-)

**COC 13**	F	(-)	7 (T4)	16 (T8)	(-)	(-)	13

**COC 14**	M	(-)	(-)	(-)	(-)	(-)	(-)

**COC 15**	F	(-)	(-)	(-)	(-)	(-)	(-)

**COC 16**	M	ND	(-)	(-)	(-)	ND	ND

**COC 17**	M	(-), bgd	(-), bgd	14 (T8), bgd	6	(-)	5

**COC 18**	F	(-), bgd	(-), bgd	(-)	(-)	(-)	(-)

**COC 19**	M	(-)	(-)	(-)	(-)	(-)	1087

**COC 20**	M	(-)	(-)	5(T8)	(-)	(-)	(-)

* (-) = absence, or xx = presence of antigen-specific proliferation. (T4) or (T8) stands for the nature of the proliferating lymphocyte population, and the number for specific antigen to control antigen ratio. The CEF panel of EBV, CMV and Flu peptides is described in ref 12.

** (-) = no antigen specific ELISPOT; xx = presence of antigen specific ELISPOT response. The number stands for specific antigen to control ratio.

Abbreviations: *bgd *background level in absence of antigen; *ND *not determined.

**Table 3:** Responses in uninfected volunteers at risk for exposure to HCV

I.D	Sex	Proliferation*			Elispot**		
		
		Core	NS3	CEF	Core	NS3	CEF
**CIC 03**	F	(-), bgd	(-), bgd	(-), bgd	(-)	(-)	38

**CIC 04**	F	(-)	(-)	5(T8)	(-)	(-)	(-)

**CIC 05**	F	(-), bgd	(-), bgd	43(T8), bgd	25	(-)	572

**CIC 06**	F	(-)	(-)	(-)	(-)	(-)	(-)

**CIC 07**	M	(-), bgd	(-), bgd	(-), bgd	(-)	(-)	(-)

**CIC 08**	F	(-)	(-)	47(T8)	(-)	(-)	47

**CIC 20**	M	bgd	bgd	bgd	(-)	(-)	(-)

**CIC 22**	M	(-)	(-)	18 (T8)	344	ND	ND

**CIC 50**	M	(-)	(-)	(-)	(-)	(-)	(-)

**CIC 54**	F	(-)	(-)	306(T8)	(-)	(-)	(-)

**CIC 55**	M	(-)	(-)	(-)	27	(-)	10

**CIC 58**	F	(-)	(-)	(-)	(-)	(-)	(-)

**CIC 59**	M	(-)	(-)	(-)	39	ND	ND

**CIC 62**	F	(-)	(-)	(-)	(-)	(-)	429

**CIC 63**	F	(-)	(-)	(-)	(-)	(-)	13

**EFS 01**	F	(-)	(-)	(-)	(-)	(-)	33

**EFS 02**	M	(-)	(-)	(-)	(-)	(-)	7

**EFS 03**	F	(-)	(-)	(-)	(-)	(-)	(-)

**EFS 06**	F	(-)	(-)	120(T8)	(-)	(-)	44

**EFS 07**	F	(-)	(-)	(-)	(-)	(-)	(-)

**EFS 09**	F	(-)	(-)	8(T8)	(-)	(-)	6

**EFS 11**	F	7(T4)	(-)	5(T8)	(-)	(-)	29

**EFS 12**	F	(-)	(-)	110(T8),11(T4)	(-)	bgd	bgd

**EFS 13**	F	(-)	(-)	(-)	(-)	(-)	(-)

**EFS 14**	F	(-)	10(T4)	8(T8)	(-)	(-)	178

**EFS 15**	F	(-)	(-)	(-)	(-)	(-)	28

**EFS 17**	F	(-)	(-)	5(T8)	(-)	(-)	63

**EFS 19**	M	(-)	(-)	15(T8)	(-)	(-)	76

**EFS 21**	F	(-)	7(T8)	21(T8)	(-)	(-)	(-)

**EFS 23**	F	(-), bgd	(-), bgd	(-), bgd	(-)	(-)	7

**EFS 24**	F	(-)	6(T8), 7(T4)	9(T8)	(-)	(-)	20

**EFS 25**	F	(-)	(-)	(-)	(-)	(-)	32

* (-) = absence, or xx = presence of antigen-specific proliferation. (T4) or (T8) stands for the nature of the proliferating lymphocyte population, and the number for specific antigen to control antigen ratio. The CEF panel of EBV, CMV and Flu peptides is described in ref 12.

** (-) = no antigen specific ELISPOT; xx = presence of antigen specific ELISPOT response. The number stands for specific antigen to control ratio.

Abbreviations: *bgd *background level in absence of antigen; *ND *not determined.

**Figure 2:**
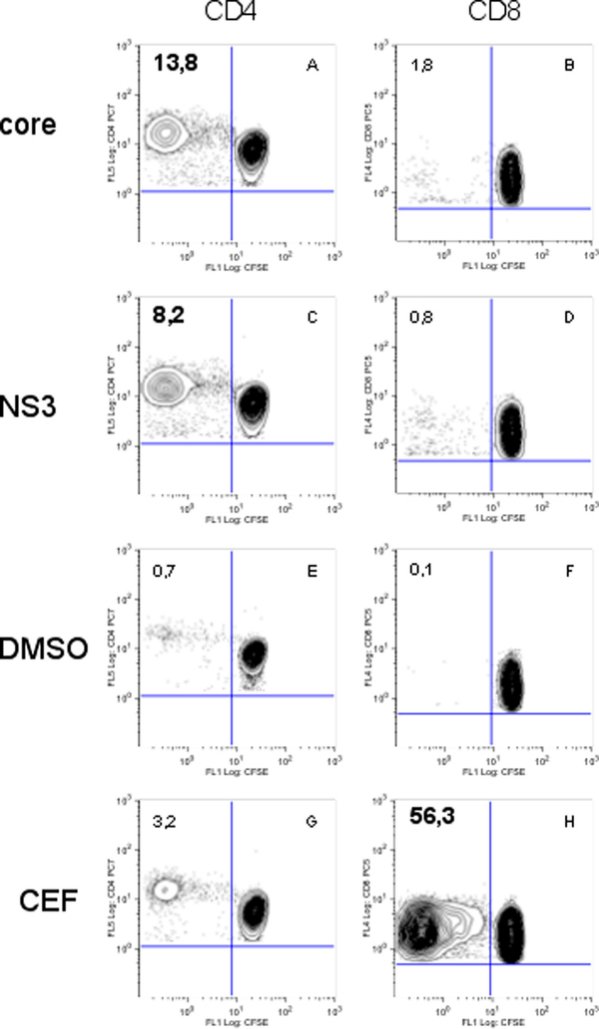
**example of proliferation for PBMC of the chronically infected CIC 38 volunteer**. Dot-plots show the percentage of proliferative CD8 ^+ ^(FL1/FL4) or CD4 ^+ ^(FL1/FL5) - T cells. The number in the upper left panel stands for the percentage of CD4^+ ^or CD8^+ ^proliferative cells among the total CD4^+ ^or CD8^+ ^-T lymphocyte population, respectively, in the absence (DMSO) or the presence of Core, NS3 or CEF antigens. The positive responses are in bold characters.

In the group of chronically infected patients (CI) 17 patients were tested (Table 1), and three showed a Core-specific response: one (CIC 34) involving the CD8 population and two (CIC 38, 46) the CD4 population (Table 4, and Figure 2, panel A). For NS3, 2/16 patients were positive: one involving CD8 cells (CIC18), and one CD4 (CIC 38) (Table 4, and Figure 2 panel C). Thus one patient (CIC 38) was positive for both NS3 and Core, in both cases the response involving the CD4 population.

**Table 4:** Characteristics of the 20 pairs of chronically HVC infected patients and their exposed uninfected partners

I.D*		Sex	HCV infection**				Proliferation***			Elispot****		
			
			Viral load	Genotype	Duration	Mode	Core	NS3	CEF	Core	NS3	CEF
**CI**	**CIC 02**	F	354 000	1b	22	Blood T	(-)	(-)	143(T8)	10	ND	ND
**EUI**	**CIC 01**	M	-	-	-	-	(-), bgd	(-), bdg	10(T8), bgd	(-)	(-)	4

**CI**	**CIC 10**	F	20 000	4	ND	Unknown	(-)	(-)	18(T8)	(-)	(-)	6
**EUI**	**CIC 11**	M	-	-	-	-	ND	ND	ND	(-)	bgd	bgd

**CI**	**CIC 12**	F	210 000	4c/b	15	Surgery	bgd	bgd	bgd	(-)	ND	ND
**EUI**	**CIC 13**	M	-	-	-	-	(-), bgd	(-), bgd	5(T8), bgd	(-)	(-)	16

**CI**	**CIC 14**	F	1 850 000	1a	24	IVDU	(-)	(-)	11(T8)	(-)	ND	ND
**EUI**	**CIC 15**	M	-	-			ND	ND	ND	(-)	(-)	13

**CI**	**CIC 18**	F	380 000	3	24	IVDU	(-)	76 (T8)	(-)	21	295	(-)
**EUI**	**CIC 19**	M	-	-	-	-	(-)	(-)	(-)	(-)	(-)	(-)

**CI**	**CIC 25**	M	1 300 000	1b	23	tattooing	(-)	ND	ND	4	ND	ND
**EUI**	**CIC 24**	F	-	-	-	-	(-)	(-)	83(T8)	12	ND	ND

**CI**	**CIC 26**	M	215 000	1b	16	Blood T	(-)	(-)	11(T8)	(-)	ND	ND
**EUI**	**CIC 27**	F	-	-	-	-	(-), bgd	(-), bgd	(-), bgd	(-)	ND	ND

**CI**	**CIC 29**	F	30 000	ND	ND	Unknown	bgd	bgd	bgd	(-)	(-)	(-)
**EUI**	**CIC 28**	M	-	-	-	-	(-)	(-)	37(T8)	7	bgd	bgd

**CI**	**CIC 31**	M	200 000	1b	37	Blood T	ND	ND	ND	8	ND	ND
**EUI**	**CIC 30**	F	-	-	-	-	(-)	(-)	9(T8)	55	(-)	45

**CI**	**CIC 32**	M	1 425 000	1b	ND	Unknown	(-)	(-)	(-)	10	ND	ND
**EUI**	**CIC 33**	F	-	-	-	-	(-)	(-)	(-)	(-)	ND	ND

**CI**	**CIC 34**	M	100 000	1b	16	Blood T	14(T8)	(-)	(-)	(-)	ND	ND
**EUI**	**CIC 35**	F	-	-	-	-	(-)	(-)	(-)	(-)	(-)	89

**CI**	**CIC 36**	F	35 000	3a	ND	Unknown	(-)	(-)	29(T8)	(-)	ND	ND
**EUI**	**CIC 37**	M	-	-	-	-	(-)	(-)	(-)	12	(-)	72

**CI**	**CIC 38**	F	260 000	1a	43	Blood T	7(T4)	4(T4)	433(T8)	(-)	ND	ND
**EUI**	**CIC 39**	M	-	-	-	-	(-)	(-)	(-)	(-)	ND	ND

**CI**	**CIC 40**	M	332 000	1a	16	Blood T	(-)	(-)	10(T8)	19	8	100
**EUI**	**CIC 41**	F	-	-	-	-	(-)	(-)	(-)	10	ND	ND

**CI**	**CIC 42**	F	140 000	1b	20	Blood T	(-)	(-)	(-)	(-)	(-)	(-)
**EUI**	**CIC 43**	M	-	-	-	-	(-)	(-)	(-)	(-)	7	(-)

**CI**	**CIC 44**	F	82 000	1b	22	Blood T	ND	ND	ND	(-)	ND	ND
**EUI**	**CIC 45**	M	-	-	-	-	(-)	(-)	(-)	11	(-)	(-)

**CI**	**CIC 46**	M	2 200 000	1b	25	Blood T	6(T4)	(-)	434(T8)	10	(-)	2070
**EUI**	**CIC 47**	F	-	-	-	-	(-)	(-)	(-)	(-)	ND	ND

**CI**	**CIC 51**	M	180 000	2a/c	22	Blood T	ND	ND	ND	(-)	ND	ND
**EUI**	**CIC 52**	F	-	-	-	-	ND	ND	ND	(-)	ND	ND

**CI**	**CIC 60**	M	720 000	1a	18	IVDU	(-)	(-)	7(T8)	(-)	ND	ND
**EUI**	**CIC 61**	F	-	-	-	-	(-), bgd	(-), bgd	(-), bgd	(-)	ND	ND

**CI**	**CIC 64**	M	1 080 000	1b	ND	Unknown	(-)	(-)	(-)	81	ND	ND
**EUI**	**CIC 65**	F	-	-	-	-	(-)	(-)	(-)	(-)	ND	ND

*: Chronically HCV-infected subjects (CI); Exposed uninfected partners of chronically infected individuals (EUI).

**: Viral load expressed as LU per mL; Duration of HCV infection in years; *Blood T *blood transfusion, *IVDU *intravenous drug users.

** (-) = absence, or xx = presence of antigen-specific proliferation. The number xx stands for specific to control antigen ration, and (T4) or (T8) for the nature of the proliferating lymphocyte population.

***(-) = no antigen specific ELISPOT; xx = presence of antigen specific ELISPOT. The number stands for specific antigen to control ratio.

Abbreviations: *bgd *background proliferation without antigen. *ND *Not determine.

The CEF panel of EBV, CMV and Flu peptides is described in ref 12.

None of the 17 exposed but uninfected (EUI) volunteers was positive for either Core or NS3 (Table 1).

No statistical difference was found in the frequencies of proliferative responses to both HCV antigens in pair-wise comparisons of all groups (*t*-test for independent samples; all *p*'s > 0.05) (Table 1).

#### Elispot responses

The same antigens were used in Elispot assays to assess the occurrence/frequency of HCV-specific circulating effector T lymphocyte responses. Eight of 65 uninfected (UI) volunteers tested were positive for Core, and none for NS3 (Table 1). Four of the 8 volunteers that gave a positive Core response were at risk (CIC 05, 22, 55, 59; Table 3), and 4 were without any known risk for HCV exposure (CIC 16 and 17, COC 17, and EFS 20; Table 2). This last donor was also positive in the proliferation test (see above).

All 20 chronically-infected (CI) subjects were tested for IFN-γ production in response to Core, and six for IFN-γ production in response to NS3 (Table 1). Eight presented a Core-specific response (CIC 2, 18, 25, 31-32, 40, 46, 64) and two NS3-specific IFN-γ response (CIC 18 and 40). Thus 2 individuals (CIC 18 and 40) demonstrated IFN-γ production in response to both viral proteins tested (Tables 1 and 4).

Six of 20 exposed but uninfected (EUI) partners demonstrated IFN-γ production in response to Core (CIC 24, 28, 30, 37, 41 and 45), and 1 of 11 tested an IFN-γ response to NS3 (CIC 43); none responded to both viral antigens (Tables 1 and 4).

The frequencies of elispot response to Core were higher in the CI group compared to UI no risk (*p *= 0.04) and to UI at risk (*p *= 0.04). The frequencies of response to NS3 were higher in the CI group compared to UI no risk (*p *< 0.03) and to UI at risk (*p *< 0.03) (Table 1).

### CEF-specific responses

EBV, CMV and Flu are viruses commonly encountered by humans. They encode peptides corresponding to CD8 class I restricted epitopes. A CEF panel of MHC class I restricted viral peptides presented by the most common Caucasian HLA types has been described [12]. CEF represents a unique peptide pool that can be used as a positive control of antigen specific T-cell receptor-driven activation in both Elispot and proliferation assays.

Twenty-four of the 62 uninfected (UI) volunteers tested (39%) were positive for proliferation, including 14/32 volunteers at risk (44%) and 10/30 volunteers without any known risk of exposure (33%) (Table 1). For 23 volunteers the CEF-specific proliferation involved the CD8 population, and for 1 (EFS 12) both CD4 and CD8 populations (Tables 2 and 3). An example of CD8 response is shown in Figure 1 (panel F).

In sixteen chronically infected (CI) subjects tested, CEF-specific proliferation was detected in 56% (9/16) individuals (Table 1). The response was detected within the CD8 lymphocyte subset only (Table 4). Proliferation profile demonstrated by volunteer CIC38 is given as an example (Figure 2, panel H).

Five of the 17 (29%) exposed uninfected (EUI) subjects (associated with the chronically infected individuals) tested were positive, the response also being solely by CD8 T cell population (Tables 1 and 4).

A high spontaneous background involving CD4 or both CD4 and CD8 subpopulations was observed for 8/62 uninfected (UI), 2/16 chronically infected (CI), and 4/17 exposed but uninfected (EUI) subjects (Tables 2, 3 and 4).

In case of sufficient number of cells, CEF-specific responses were assessed also by IFN-γ Elispot. IFN-γ response to stimulation with CEF pool was detected in 53% (31/58) of uninfected (UI) volunteers, namely in 60% (18/30) of subjects at risk and 46% (13/28) individuals with no known risk of HCV exposure. Similarly, 50 and 55% response to CEF was detected respectively among chronic HCV (CI) carriers (3/6) and their uninfected (EUI) partners (6/11; Table 1). In summary, a high proportion of individuals in each of the groups tested positive for a CEF-specific response.

No statistical difference was seen in the frequency of CEF-specific responses registered by either proliferation or Elispot (all *p *values > 0.05). This reflected similar antigen-specific T-cell receptor driven T cell activation by HCV-unrelated antigens in all groups.

## Discussion

We have studied three groups that differed with respect to the degree of exposition to HCV: 1) chronic HCV carrier (CI); 2) individuals who remained noninfected despite repeated exposure through sexual contact contact with these carriers (EUI); 3) healthy apparently unnexposed volunteers (UI). Specific cellular immune responses against HCV Core or NS3 were assessed by Elispot or T cell proliferation assays.

The highest proportion of HCV-specific response was observed among chronically-infected subjects (Table 1). The fact that such a response was detected in less than half of the patients is in agreement with previous reports on HCV T-cell responses in chronic HCV infection [13]. Elispot responses were detected the most frequently (in 8/20 chronically-infected volunteers tested for Core, and in 2/6, tested for NS3 responses) whereas only few chronically-infected individuals presented Core or NS3-specific proliferative reactions (3/17, and 2/16, respectively).

The synthetic peptides used to screen cellular responses to HCV represent sequence of HCV genotype 1 since the majority of individuals were infected by a genotype 1 virus. Two of the three chronically-infected subjects who gave positive NS3- responses were infected with a genotype 1 virus. Seven of the eight chronically-infected subjects who were positive for Core by the Elispot assay, were also infected by HCV genotype 1. Meanwhile, chronically-infected patients carried also HCV of three other genotypes: one was infected by a genotype 2 virus, and two by each of genotypes 3 and 4 (Table 4). Very low frequency of NS3-responders amongst patients infected with HCV genotype 2, 3 or 4, may reflect a limited number of non-HCV genotype 1 infected individuals included in this study and also the genotype variation of NS3 sequence. The latter explanation is, however, hardly applicable for the core-specific responses, since HCV core is highly conserved with very few amino acid inter-genotype differences [14].

The low frequency of proliferative responses compared to Elispot could be attributed to a higher sensitivity of the latter (assay). However, in our view, decisive is the type of the registered response. Elispot assays performed with *ex vivo *isolated PBMC preferentially detect effector lymphocytes that do not need to expand, while assays using *in vitro *expanded T lymphocytes rather detect precursors of memory T cells with a proliferative capacity [15]. The low frequency of proliferative responses among chronic HCV carriers may rather reflect a weak HCV memory response (specifically when comparing chronic hepatitis C patients to those resolving HCV infection; for review, 13). Of particular note, relatively few individuals gave a concomitant positive response in both assays. This absence of correlation between Elispot and proliferative responses in chronically-infected individuals suggests that effector and memory T cells are distinct T cell populations, probably recognizing different epitopes. Such phenomenon was described earlier [16].

Interestingly, HCV Core-specific Elispot responses were observed in a relatively high proportion (30%) of the uninfected partners of chronically-infected individuals (Table 1). This is in agreement with previous reports on the populations of uninfected seronegative individuals exposed to HCV, including healthy relatives of HCV-infected individuals, intravenous drug users, and individuals with occupational exposure [17-21].

The most striking result of the current study was that despite stringent criteria of the positive cellular response, an HCV-specific response was registered in 20% of uninfected subject tested (13/65; Tables 2 and 3). This group was split into two subgroups, depending on the possibility of exposure to HCV. Indeed, eight individuals who displayed a positive result could have been exposed to HCV (professionally), although there was no clear history of contamination (Table 3). No such risk was, however, identified to explain positive results in the remaining five individuals (Table 2).

The detection of HCV specific cellular responses in uninfected volunteers reflects the difficulty to precisely identify all (possible) risks of exposure to HCV. Furthermore, it may also reflect a past inapparent HCV infection. Clearance of HCV viremia associated with cellular immunity in the absence of seroconversion has been reported in populations at risk for HCV exposure [5,22,23].

Other causes for detecting HCV-response in healthy risk-free individuals cannot be categorically ruled out. Two uninfected volunteers had positive proliferative response for Core: one with no risk of exposure to HCV (EFS 20) gave a response that involved both CD4 and CD8 populations, whereas the other that only implicated a CD4 population response (EFS 11) was retrospectively shown to have been exposed to HCV. For EFS 20, we could map the reactive sequence to Core amino acid residues 173-190 (not shown). An extensive sequence search using the BLAST tool [24] revealed a eight amino acid homology between HCV Core 174-185 (FSIFLLALLSCL) and HBs antigen 41-52 (FIIFLFXLLXCL). While it remains possible that the observed reactivity corresponds to a cross-reactive immunization [8-10], it is noteworthy that EFS 20 was neither infected nor immunized with HBV.

NS3-specific proliferative responses were observed in four uninfected volunteers (COC 13, and EFS 14, 21 and 24). The PBMC of these individuals were also reactive to the CEF peptides including 12 influenza epitopes. As immunization against Influenza virus neuraminidase was reported to generate immune responses crossreactive with HCV NS3 [10], we cannot formally exclude that T-cell proliferation in response to NS3 resulted from cross-reactivity.

In all three groups, a much higher proportion of individuals tested positive for CEF-specific response registered by Elispot and proliferation tests. The proportion of responders (number of positive/total number tested) varied between groups, but was within the limits of stochastic variations: between 56% (9/16) for the chronically-infected subjects and 29% (5/17) for their uninfected partners (in proliferation). These figures matched the range of proportions seen in CEF-positive Elispots: 60% (18/30) for at risk uninfected volunteers and 46% (13/28) for uninfected volunteers with no known risk of HCV-exposure (Table 1). This was somewhat lower that the data reported by Currier et al. [12], but similar to that reported by Horton et al. [25] possibly reflecting the heterogeneity of the HLA alleles in the studied groups. All CEF-specific proliferative responses involved the CD8 subpopulation, and in 1 of 95 individuals, both the CD8 and CD4 compartments. This is not surprising since most of the CEF peptides were 8 to 9 mers representing CD8 class I-restricted epitopes, although CD4-specific cytotoxic responses have also been reported in human viral infections [26-28].

As there were no statistical difference between the groups in the frequencies of proliferative or Elispot responses to the control (CEF) antigens (Table 1), exposure to or infection by HCV did not seem to have any major impact on the frequency of cellular responses to unrelated viruses. Hence, it is unlikely that the number of positive cellular responses to HCV antigens could be explained by antigen stimulation(s) specific to other viral antigens. In addition, pair-wise comparisons revealed no difference in the occurrence of cellular immune response against HCV core and/or NS3 among CEF-negative versus CEF-positive individuals in any of the groups (UI with known risk, UI at risk, EUI, or CI; all *p *values > 0.3). Thus, there is no evidence demonstrating that anti-CEF cellular reactivity interfere with the detection of anti-HCV cellular responses.

Alternatively, atypical HCV-specific immune responses may be generated by the occult HCV infections of the liver [29]. Such infections have been described for patients with abnormal liver function of unknown origin, who present negative HCV PCR and Elisa results in the serum but where HCV RNA is detected in the liver [30]. However, in our study, all uninfected CIC volunteers had normal liver biology. For the twenty COC individuals, liver function was investigated using the Fibrotest [31], and all gave a normal value (not shown). Thus, it is likely that, in this study, the detection of a positive HCV-specific cellular response did not reflect an occult HCV infection.

The polymorphism of the IL28B gene has been recently associated with both spontaneous resolution of HCV infection and sustained virologic response in pegylated interferon/ribavirin treated patients [32-34]; we can speculate that such a polymorphism may explain partially our results but this study was initiated before the first report and we are unauthorized to make a retrospective genetic study.

In summary, the detection of HCV-specific immune responses in uninfected volunteers may reflect an under-estimated prevalence of inapparent and resolving acute HCV infections. This changes our understanding of the epidemiology and the physiopathology of HCV infection. An alternative, not mutually exclusive, hypothesis is the existence of cross-reactivity between HCV antigens and other viral or common antigens present in the general population, as previously suggested by other researchers.

### Patients and methods

#### Patients and volunteers

Sixty-five presumably unexposed and uninfected volunteers (Uninfected individuals, UI) were studied. All volunteers were negative in HCV PCR assay (ABBOTT Real Time HCV, Abbott, Rungis, France, threshold < 12 I.U/ml) and had a negative HCV-specific humoral response according to a commercial Elisa assay (MONOLISA anti-HCV Plus V2, Biorad, Marnes-la-Coquette, France). This enzyme immunoassay contains HCV recombinant proteins expressed in E coli including sequences from NS3 and NS4 and from the structural core protein. All volunteers were not infected by HBV or HIV. These volunteers were categorized according to the putative risk of exposure to HCV [no known risk (n = 33), Table 2; at risk (n = 32), Table 3]. Exclusion factors for exposure to HCV [11] were: professional exposure, drug abuse, blood transfusion or injection of blood products, sexually transmitted diseases, incarceration, alcoholism, dialysis, endoscopy, acupuncture, mesotherapy, invasive cosmetic treatment, piercing, tattooing, sexual exposure, familial exposure, and hospitalization or outpatient treatment in a developing country. The 65 volunteers were recruited in three distinct centers located in the Paris area. Initialy enrolled was a group of 20 uninfected volunteers [mean age: 46 year; range: 27-65; sex ratio: 1] (Necker Clinical Investigation Center, CIC volunteers). However, it was retrospectively reported that fifteen individuals from this group might have been exposed to HCV due to their occupational status. A second group of 25 volunteers was recruited at a french blood center in Paris (Etablissement Français du Sang (EFS), Paris; EFS 01 to 25). This group comprised 8 volunteers without any known risk for exposure to HCV [mean age: 27.8 year; range: 18-40; sex ratio: 0.14] and 17 volunteers at risk [mean age: 43.1 year; range: 21-64; sex ratio: 0.13]. The third group of 20 volunteers with no known risk for exposure to HCV was recruted at the Center for clinical investigation of the Cochin Hospital, Paris [COC 01 to 20; mean age: 27.4 year; range: 18-41; sex ratio: 1.2].

Twenty chronic HCV infected carriers and their exposed uninfected sexual partners were included as positive controls for HCV infection and potential exposure, respectively (Table 4). Infected patients [mean age: 46 year; range: 24-66; sex ratio: 1] were all HCV seropositive and viraemic. All viruses were genotyped except for one volunteer; the HCV genotypes were: 1b (n = 10), 1a (n = 4)], 2a/c (n = 1), 3 (n = 2), and 4 (n = 2). The mode of contamination was established for fifteen individuals; ten were infected by blood transfusion, one after surgery, one following a tattooing procedure, and three were intravenous drug users. The 20 exposed uninfected partners [mean age: 44 year; range: 26-63; sex ratio: 1] were active sexual contacts (> 2 years) of these infected HCV carriers. All exposed uninfected individuals were HCV seronegative and HCV-RNA negative by PCR.

None of the volunteers was infected by HIV, and all had a normal blood cell count the day of harvesting PBMCs. Biomedical research was approved by the local ethics committee (RBM 01-24), and was carried out in accordance with the Helsinki Declaration.

#### Preparation of PBMC

PBMCs were isolated from heparinized blood as described [35]. The PBMCs were frozen at -80°C in 90% fetal calf serum (D. Dutscher, Strasbourg, France) containing 10% DMSO (Pierce, ThermoFisher, Brebières, France), and stored in liquid nitrogen until used.

#### Synthetic peptides

The consensus sequence of the Core protein (genotype 1a) was covered by thirty-seven 15 mer peptides that overlapped by 10 residues, as described [35]. NS3 [consensus 1b, aa 1027-1657] was represented by sixty-eight overlapping 15 mer peptides corresponding to regions encoding the CD4 and CD8 epitopes were used. These clusters of T4 and T8 epitopes corresponded to the following regions: aa 1072-1111 (TCVN... LVGW); 1167-1191(GPLL... GVAK); 1199-1355(SMET... TDAL); 1461-1475 (TVDF... IETT); 1531-55(TPAE... QDHL); 1576-1652(TQKA... ACMS), according to the Los Alamos databases [36,37]. A pool of unrelated 12-to 15-mer peptides derived from Gag and Nef of simian immunodeficiency virus (SIVmac239) were used as a negative control. Core and SIV peptides were purchased from NeoMPS (Strasbourg, France), and NS3 ones from Proimmune (Oxford, UK). Each peptide was certified to be > 80% pure, by RP-HPLC. Positive control was a pool of 32 peptides (CEF) corresponding to well-characterized CD8 class I restricted epitopes of human cytomegalovirus (CMV), Epstein-Barr virus (EBV) and Influenza virus [12]. CEF pool was obtained through the NIH AIDS Research and reference reagent program, or Anaspec Inc., San Jose, CA, USA. The peptides were dissolved in DMSO at 1 mg/ml, and were stored at -80°C until used.

#### Immunological assays

Virus-specific circulating effector T lymphocyte responses were studied using two distinct functional assays:

##### Elispot assay

HCV-specific T cell responses of freshly isolated or frozen PBMC were studied by *ex vivo *ELISPOT assays [38], using the panels of Core or NS3 peptides described above. Peptides were used at a final concentration of 1 μg/ml. Negative controls consisted of cells incubated in medium. Phorbol myristate acetate and ionomycin (25 and 100 ng/ml, respectively; Sigma-Aldrich Chimie, Saint-Quentin Fallavier, France) were used as positive controls. The frequencies of IFN-γ producing cells were expressed as the number of spot-forming cells (SFC) per 10^6 ^cells. Frequencies lower than 50 spots/l million PBMC were considered unspecific. An assay was considered positive if: 1/. The number of spots generated in response to stimulation with specific peptides exceeded the mean of the number of spots obtained with culture medium plus 2 SD; and 2/. Its ratio to the number of spots with culture medium was > or = 4.

##### Proliferation assay

PBMC (2 ×10^6^/ml) were labelled with 10 mM carboxyfluorescein diacetate succinimidyl ester (CFDA-SE; Invitrogen, ref C1157) in serum-free medium for 30 min at 37°C [39]. Labeled PBMC were washed with complete medium (D-MEM +1% non essential aminoacids, 1 mM L-glutamine, Invitrogen, Cergy, France) supplemented with 10% heat inactivated human AB serum (SAB, Biowest, France), and incubated in complete D-MEM culture medium at 37°C under 5% CO_2_. The following antigen stimulations were performed: 1/. HCV-specific with pools of Core or NS3-specific peptides each at a final concentration of 1 μg/ml; 2/. Common antigen-specific CEF peptides as positive control (final concentration 0.5 μg/ml); 3/. Mitogen (superantigen) Staphylococcus Enterotoxin B (Ref S4881, Sigma, St Louis, MI) at 500 ng/mL as positive control for PBMC viability; 4/. An irrelevant SIV-peptide pool, and complete medium plus 0.05% DMSO (peptide diluent) as negative controls.

After 6 days incubation, cells were washed in PBS and incubated for 30 min at 25°C with anti CD3 phycoerythrin-Texas Red (ECD)-, anti-CD8β phycoerythrin-cyanin 5 (PCy5)-, and anti CD4 phycoerythrin-cyanin 7 (PCy7)-conjugated monoclonal antibodies (refs A07748, 6607101, and 737660 from Beckman-Coulter respectively). At the end of the incubation period, cells were washed twice in PBS and fixed with 200 μL of 2% formaldehyde solution in PBS for 15 min at 25°C. Cell division accompanied by CFSE dilution [39] was analyzed by flow cytometry. For each sample, at least 10^5 ^events were acquired using a FC500 cytometer (Beckman Coulter). Data were analysed with FlowJo (TreeStar). Lymphocytes were gated based on their forward and side scattering dot plot. T lymphocytes were defined based on their expression of CD3 and CD4 or CD8. The following criteria for antigen-specific proliferation were set: 1/. Background of proliferation without antigen (DMSO) < 4%; 2/. Antigen proliferation ratio (Antigen/SIV) > or = 4.; 3/. Absolute number of proliferating cells (i.e. CFSE negative) > 100; 4/. Threshold value > mean of difference between control antigen (SIV) + 2 SD.

### Statistics

Frequencies of HCV-specific proliferative and IFN-γ ELISPOT responses between groups were compared between the groups pairwisely using two-sided *t*-test for independant samples assessing difference in proportions. Tests were done using Quick Calcs, Graph Pad Software.

## Abbreviations

EUI: Exposed Uninfected Individuals; UI: Uninfected Individuals; CI: Chronically Infected Individuals.

## Competing interests

The authors have no competing interests with any commercial or other association in conjunction with the research presented herein.

## Authors' contributions

TM, GJ, CM, LD carried out the elispot and proliferation assays. MLC carried out the virological assays. MLC, OL, JLB, SP participated in the design of the study and were responsible for the clinical data from volunteers. MI, TM ad JLB participated in the statistical analysis. YR was responsible for the design and coordination of the study, and for the writting of the manuscript. All authors read and approved the final manuscript.

## Authors' informations

Present address for L. Durrieu: Centre de recherche CHU Ste-Justine, Département de Microbiologie et Immunologie, Université de Montréal, 3175, Chemin de la Côte-Sainte-Catherine, Montréal H3T 1C5 Québec, Canada.

Corresponding author contact information: Yves Rivière, Viral Immunopathology URA CNRS 3015, Department of Virology, Lwoff Building, Institut Pasteur, 28 rue du Dr Roux, 75015 Paris. tel 331 4568 8778; fax 331 4061 3298; yves.riviere@pasteur.fr or yves.l.riviere@gmail.com.
